# Dickkopf‐1‐promoted vasculogenic mimicry in non‐small cell lung cancer is associated with EMT and development of a cancer stem‐like cell phenotype

**DOI:** 10.1111/jcmm.12862

**Published:** 2016-05-31

**Authors:** Lingli Yao, Danfang Zhang, Xiulan Zhao, Baocun Sun, Yanrong Liu, Qiang Gu, Yanhui Zhang, Xueming Zhao, Na Che, Yanjun Zheng, Fang Liu, Yong Wang, Jie Meng

**Affiliations:** ^1^ Department of PathologyTianjin Medical UniversityTianjinChina; ^2^ Department of PathologyTianjin General HospitalTianjin Medical UniversityTianjinChina; ^3^ Department of PathologyTianjin Cancer HospitalTianjin Medical UniversityTianjinChina

**Keywords:** DKK1, vasculogenic mimicry, cancer stem‐like cell, epithelial‐mesenchymal transition, non‐small cell lung cancer

## Abstract

To characterize the contributions of Dickkopf‐1 (DKK1) towards the induction of vasculogenic mimicry (VM) in non‐small cell lung cancer (NSCLC), we evaluated cohorts of primary tumours, performed *in vitro* functional studies and generated xenograft mouse models. Vasculogenic mimicry was observed in 28 of 205 NSCLC tumours, while DKK1 was detected in 133 cases. Notably, DKK1 was positively associated with VM. Statistical analysis showed that VM and DKK1 were both related to aggressive clinical course and thus were indicators of a poor prognosis. Moreover, expression of epithelial‐mesenchymal transition (EMT)‐related proteins (vimentin, Slug, and Twist), cancer stem‐like cell (CSC)‐related proteins (nestin and CD44), VM‐related proteins (MMP2, MMP9, and vascular endothelial‐cadherin), and β‐catenin‐nu were all elevated in VM‐positive and DKK1‐positive tumours, whereas the epithelial marker (E‐cadherin) was reduced in the VM‐positive and DKK1‐positive groups. Non‐small cell lung cancer cell lines with overexpressed or silenced DKK1 highlighted its role in the restoration of mesenchymal phenotypes and development of CSC characteristics. Moreover, DKK1 significantly promotes NSCLC tumour cells to migrate, invade and proliferate. *In vivo* animal studies demonstrated that DKK1 enhances the growth of transplanted human tumours cells, as well as increased VM formation, mesenthymal phenotypes and CSC properties. Our results suggest that DKK1 can promote VM formation *via* induction of the expression of EMT and CSC‐related proteins. As such, we feel that DKK1 may represent a novel target of NSCLC therapy.

## Background

Primary lung cancer is the leading cause of cancer mortality in the world 1. Some advances in diagnosis and treatment have made significant strides towards improving these trends; however, the survival rate of patients with lung cancer remains poor. Thus, further research is critically necessary to expand our understanding of this disease and identify novel, actionable targets. Non‐small cell lung cancer (NSCLC) comprises approximately 80% of all lung cancer cases 1, 2.

In 1999, Maniotis *et al*., detected a novel mode by which highly aggressive uveal melanoma maintain blood supply. Specifically, the authors identified blood vessels that are completely surrounded by tumour cells, a condition that was designated vasculogenic mimicry (VM) 3. Since the discovery of VM, research has focused on the molecular mechanism by which VM formation occurs in an effort to identify actionable targets. Epithelial‐mesenchymal transition (EMT) and cancer stem‐like cell (CSC) have been associated with the formation of VM in several tumours 4, 5, 6. As the Wnt signalling pathway – which provides important clues to embryonic development and tumorigenesis – is implicated in both EMT and CSC, we evaluated whether this pathway participates in VM 7, 8.

Dickkopf‐1 (DKK1), which plays a crucial role in head formation in vertebrate development, encodes a secreted protein and is a negative regulator of the Wnt signalling pathway in numerous cancers 9, 10. Previous studies have reported that overexpression of DKK1 is negatively correlated with the existence of VM, and may reduce proliferation, migration and invasion of colon cancer cells 8. By contrast, DKK1 exhibits a potential oncogenic function as a result of elevated expression detected in Wilms' tumour, hepatoblastoma and hepatocellular carcinoma (HCC) 11, 12. A previous report has shown that DKK1 is also highly expressed in NSCLC, and may be useful as a novel diagnostic and prognostic marker for lung cancer 13.

However, the relationship between DKK1 and VM in NSCLC remains unknown. In this study, we aimed to identify the potential contribution of DKK1 in the formation of VM. We hypothesized that DKK1 promotes VM formation *via* induction of EMT and development of CSC characteristics. To evaluate or premise, we obtained large cohorts of human NSCLC tissues to identify the clinical and biological overlap between VM and DKK1 expression. Subsequently, cell culture and xenograft mouse models were used for *in vitro* and *in vivo* studies, respectively.

## Materials and methods

### Patients

Tissue specimens were obtained from 205 patients who had undergone surgical resection for lung cancer in Tianjin Medical University Cancer Institute and Hospital from October 1990 to November 2010. These 205 NSCLC samples included 79 cases of squamous cell carcinoma, 75 cases of adenocarcinoma and 51 cases of large cell cancer. The diagnoses of these samples were verified by two pathologists according to the standards of classification 2, 14. Clinicopathological parameters were obtained from patients' clinical records and pathological reports. Total survival time, final follow‐up examination and diagnosis of metastasis were recorded from the date of surgery. This study was approved by the Ethical Committee of Tianjin Medical University.

### Immunofluorescence, immunohistochemistry and CD31/periodic acid Schiff double‐staining

Immunohistochemistry was performed as described by Sun *et al*. 4, 15. The details of the primary antibodies are listed in Table S2. Secondary antibodies were purchased from Zhongshan Golden Bridge (Beijing, China). Positive and negative controls were run for each batch. PBS was used as substitute for primary antibodies in the negative controls. The results were evaluated according to the method described by Bittner *et al*. 16.

### Cell culture and plasmid transfection

Human NSCLC cell lines H1299, H460 and A549 (Cell Resource Center, Institute of Basic Medical Sciences, Chinese Academy of Medical Sciences, School of Basic Medicine, Peking Union Medical College) were cultured in RPMI‐1640 with 10% foetal bovine serum (FBS; Invitrogen, Grand Island, NY, USA). Transfection was performed with Lipofectamine 2000 (Invitrogen, Carlsbad, CA, USA) using a plasmid carrying DKK1 o empty vector controls, purchased from Genechem Company (Shanghai, China). Briefly, DKK1 cDNA was amplified using the following primer sequences: forward primer: 5′‐CTAGCTAGCACATGATGGCTCTGG‐3′ and reverse primer: 5′‐GGAATTCGTGTCTCTGACAAGTGTG‐3′, digested with XhoI/EcoRI and subcloned into pcDNA3.1 vectors. The small interfering RNA kit (HSH005612‐mH1 H1 Puromycinmcherry psi‐mH1) was purchased from GeneCopoeia (Guangzhou, China). Transfection was performed with Lipofectamine 2000 (Invitrogen). G418 was used to select clones to establish stable H460 cells that overexpressed DKK1 and A549 cells with reduced expression of DKK1.

### Western blot analysis

Western blot was performed according to previously published protocols 13. A monoclonal beta‐actin antibody (Santa Cruz, Dallas, TX, USA) was used for protein loading analysis, and the other primary antibodies are described above. Secondary antibodies were also purchased from Santa Cruz Biotechnology.

### Cell proliferation assay

Cells were cultured in 96‐well plates at a concentration of 1 × 10^3^ cells per well and incubated for various periods (1, 2, 3, 4, 5 and 6 days). At a specified time, MTT (Sigma‐Aldrich, St. Louis, MO, USA) solution was added. After 4 hrs incubation at 37°C, dimethyl sulfoxide was used to dissolve the purple crystals by shaking for 10 min. on a table concentrator. The optical density was determined at 490 nm on Spectra Max M2 (Molecular Devices, Sunnyvale, CA, USA).

### Invasion assay

Cell invasion was detected using transwell cell culture inserts with 8 μm membrane pore size (BD Biosciences, San Jose, CA, USA). Matrigel (2 mg/ml; BD Biosciences) was placed on the upper surface of the chamber. Subsequently, 200 μl cell suspension (2 × 10^5^ cells/ml) without bovine serum was added to the upper chamber, and 500 μl RPMI‐1640 containing 20% FBS was added to the lower chamber. Following incubation at 37°C with 5% CO_2_ for 48 hrs, the passed cells were fixed, stained and counted. Each experiment was performed in triplicate.

### Migration and wound‐healing assay

Migration assay was performed in the same manner as an invasion assay, except that the inner surface was not supplied with Matrigel and the incubation time was 24 hrs. For the wound‐healing assay, cells were placed in a 12‐well plate to form a monolayer 1 day before the assay. After making a uniform straight scratch with a pipette tip, cells were incubated at 37°C with 5% CO_2_, and cell motility was assessed by measuring the speed of wound closure at intervals. Each experiment was performed in triplicate.

### 3D culture assay

This assay was conducted as previously described 4, 15. The cells on Matrigel were pictured with a microscope (TS100‐F; Nikon, Shinagawa‐Ku, Tokyo, Japan). Six pictures in each group were collected, and the number of VM channels was counted.

### Animal studies

A total of 20 male BALB/c nude mice, 3‐ to 4‐weeks of age (purchased from Beijing H\FK Bioscience Co., Ltd., Beijing, China), were divided randomly into four groups: H460‐control group, H460‐transfection group, A549‐control group and A549‐transfection group. Each mouse was subcutaneously injected in the right armpit with 5 × 10^6^ cells. The tumour size was measured every 3 days for 21 days with sliding calliper. The tumour volume was calculated using the following formula: volume = (length [mm] × width^2^ [mm])/2. Tumour samples were fixed in formalin, embedded in paraffin, slide cut (4 μm thick) and immunochemically stained. All animal experiments were conducted according to the guidelines of Tianjin Medical University, China.

### Statistical analysis

All data in the study were evaluated using SPSS17.0 software (SPSS, Chicago, IL, USA). Survival data were analysed by Kaplan–Meier analysis. C, Pearson chi‐squared test, Spearman correlation analysis and *t*‐test were used as needed. All *P*‐values were two‐sided, and *P* < 0.05 was considered a statistically significant test.

## Results

### Association of VM and DKK1 with clinicopathological features in human NSCLC samples

**Figure 1:**
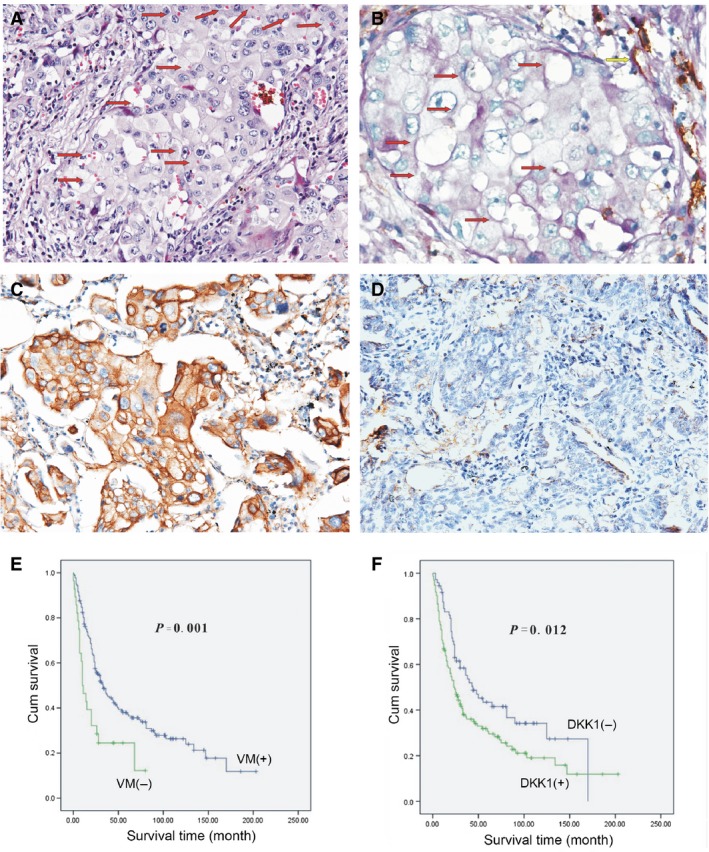
(**A**) Morphological appearance of VM with hematoxylin and eosin staining. The VM channel was surrounded by tumour cells and RBC (red arrow). Absence of necrosis and phlogocyte was observed in the vicinity (×200). (**B**) Results of CD31/PAS double‐staining (×400). The VM channel (red arrow) was PAS‐positive, but it did not expressed CD31. Endothelium‐dependent vessel (yellow arrow) was PAS and CD31‐positive. (**C**) Immunohistochemical staining for DKK1 overexpression in the VM‐positive group (×200). (**D**) DKK1 was down‐regulated in the non‐VM group (×200). (**E**) Kaplan–Meier survival analysis showing that the VM‐positive patients have shorter survival time than VM‐negative patients (*P* = 0.001). (**F**) Kaplan–Meier survival analysis showing that the DKK1‐positive patients have shorter survival time than DKK1‐negative patients (*P* = 0.012).

Based on our previous studies 4, 15, classical endothelial vessels were lined with shuttle‐like endothelium, whereas the channels surrounded by tumour cells with the presence of red blood cells were deemed VM. These channels do not display necrosis nor were infiltrating inflammatory cells observed (Fig. 1A). As shown by CD31/periodic acid Schiff (PAS) double staining, endothelium‐dependent vessels were positive for both CD31 and PAS, whereas the tumour cells lining VM channels were negative for CD31. The base membrane‐like structure between red blood cells and the tumour cells were positive for PAS (Fig. 1B). In our study, VM was detected in 28 (13.66%) of 205 specimens. The frequency of VM was significantly associated with histological classification, differentiation, T stages, clinical stages, and distant metastasis (*P* < 0.001, <0.001, 0.021, 0.001 and <0.001 respectively). Poor differentiation, high distant metastasis, and advanced stage were observed in samples in the VM‐tumours. Among the three histological types, the frequency of VM was greatest in large cell lung cancer (17/51, 33.33%), followed by adenocarcinoma (7/75, 9.33%) and squamous cell carcinoma (4/79, 5.06%). No statistically significant correlations were observed between VM and other clinicopathological features including: age, gender, tumour size, tumour location, pleural invasion, lymph node metastasis and therapy (*P* > 0.05; Table S1).

Of 205 NSCLCs tumours, 133 were DKK1‐positive (133/205, 64.88%). The specimens were also divided into two groups according to DKK1 presence. Tumour type and differentiation were significantly different between DKK1‐positive and DKK1‐negative groups (*P* = 0.028, 0.008 respectively; Fig. 1C and D), but other clinicopathological factors were not significantly associated with DKK1 expression (*P* > 0.05, Table S1). Similar to VM, DKK1 was frequently expressed in large cell lung cancer (41/51, 80.39%) and poorly differentiated lung cancer (64/83, 77.11%).

To verify the clinical significance of VM, we investigated the relationship between VM and survival outcome. The average survival period for VM‐positive patients was 25.95 months, whereas for VM‐negative patients, it is 68.07 months (Fig. 1E). The total survival period of patients with VM was significantly shorter than that of patients without VM (*P* = 0.001). In addition, Kaplan–Meier survival analysis demonstrated that DKK1 had an effect similar trend, as VM‐positive *versus* VM‐negative groups, in regard to survival. The total survival time for patients of the DKK1‐negative group was longer than that of the DKK1‐positive group (*P* = 0.012). The average survival period for patients of the DKK1‐positive group was 55.89 months, whereas that of the DKK1‐negative patients was 76.12 months (Fig. 1F).

### Relationship between VM, DKK1 and EMT/CSC/VM‐related proteins in human NSCLC tissue

To characterize the role of DKK1 in VM formation, the relationship between VM and DKK1 was examined in NSCLC tissues. Dickkopf‐1 was positively associated with VM (*P* = 0.003, *r* = 0.203). The positive expression of DKK1 in the VM group (25/28, 89.29%) was significantly higher than that in the non‐VM group (108/177, 61.02%). Dickkopf‐1‐positive and VM‐positive samples included 25 cases, and DKK1/VM‐negative samples included 69 cases. All of 205 NSCLC samples had been used in IHC staining for EMT and CSC‐related proteins.

**Figure 2:**
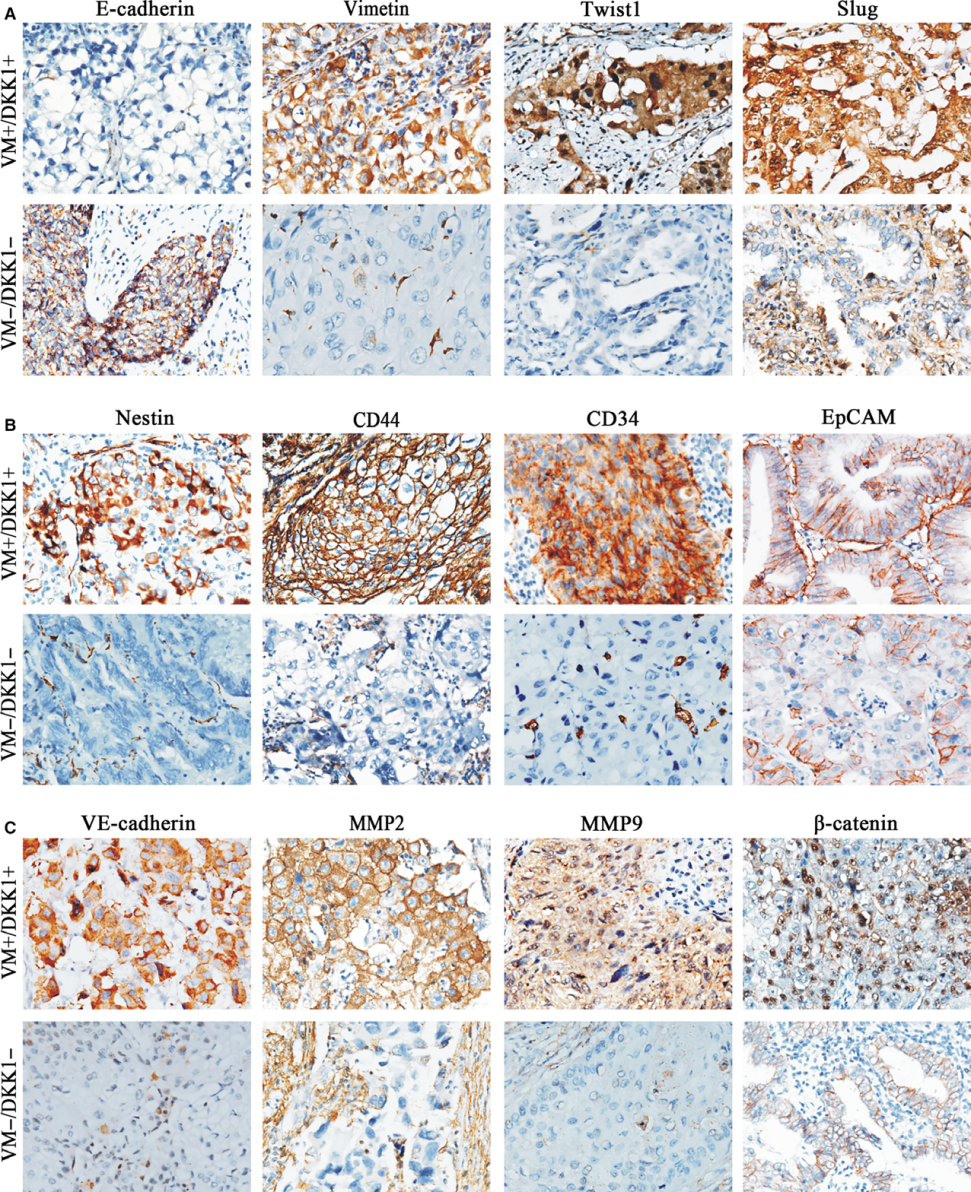
Expression of EMT and CSC‐related proteins in NSCLC (×200). (**A**) Expression of EMT‐related proteins in NSCLC. E‐cadherin was positively expressed in the membrane of tumour cells. Vimentin was considered positively expressed as it was stained in the cytoplasm of tumour cells, and the positive expression of stromal cells was considered as internal control. Positive expression of Twist was found in the cytoplasm of tumour cells, and sometimes, in the nucleus. Slug was positively located both in the cytoplasm and in the nuclei of tumour cells. (**B**) Expression of CSC‐related markers in NSCLC. Positive expressions of nestin and CD34 were both found in the cytoplasm of tumour cells. CD44 and EpCAM expressions were considered to be located in the membrane of tumour cells. Positive expressions of new blood vessels, lymphocytes and endothelial cells were considered to be internal controls for nestin, CD44 and CD34 respectively. (**C**) Expression of VM‐related proteins and β‐catenin in NSCLC. VE‐cadherin, MMP2 and MMP9 were positively expressed in the cytoplasm of tumour cells. Positive expression of β‐catenin‐nu was found from membrane to nucleus. Positive expression of VE‐cadherin in blood vessels, MMP2, and MMP9 in stromal cells were considered as internal control. These results showed samples of group, which lost their epithelial phenotypes and exhibited more mesenchymal phenotypes and CSC features.

Relationships between VM and EMT/CSC‐related proteins were also detected. As shown in Table 1, no significant relationship was found between EpCAM and VM (*P* > 0.05), whereas the differential expression levels of EMT‐related proteins (E‐cadherin, vimentin, Twist, and Slug) and CSC‐related proteins (CD44, nestin and CD34) between VM‐positive and VM‐negative groups was significant (*P* < 0.05, Fig. 2A and B). β‐catenin nuclear expression and VM‐related proteins (VE‐cadherin, matrix metalloproteinase2 (MMP2) and MMP9) were also overexpressed in the VM group (*P* < 0.05, Table 1, Fig. 2C).

**Table 1:** Correlation between VM, DKK1 and EMT and CSC‐related proteins of NSCLC

Variant	VM		*P*‐value	*r*	DKK1		*P*‐value	*r*
	Negative	Positive			Negative	Positive		
β‐catenin nuclear expression								
Negative	160	13	<0.001	0.416	68	105	0.004	0.204
Positive	17	15			4	28		
E‐cadherin								
Negative	49	19	<0.001	−0.293	17	51	0.043	−0.149
Positive	128	9			55	82		
Vimentin								
Negative	127	8	<0.001	0.313	55	80	0.021	0.163
Positive	50	20			17	53		
Twist								
Negative	126	8	<0.001	0.308	58	76	0.001	0.235
Positive	51	20			14	57		
Slug								
Negative	103	7	0.002	0.229	47	63	0.019	0.171
Positive	74	21			25	70		
EpCAM								
Negative	115	15	0.292	–	48	82	0.544	–
Positive	62	13			24	51		
Nestin								
Negative	150	18	0.015	0.183	65	103	0.023	0.159
Positive	27	10			7	30		
CD44								
Negative	83	5	0.004	0.201	39	49	0.019	0.167
Positive	94	23			33	84		
CD34								
Negative	174	25	0.035	0.008	72	127	0.093	–
Positive	3	3			0	6		
VE‐cadherin								
Negative	101	3	<0.001	0.318	53	51	<0.001	0.337
Positive	76	25			19	82		
MMP2								
Negative	125	13	0.016	0.177	55	83	0.044	0.142
Positive	52	15			17	50		
MMP9								
Negative	113	12	0.039	0.148	55	70	0.001	0.232
Positive	64	16			17	63		

The relationship between DKK1 and these related proteins was also examined. The results were similar to those of VM, with the CD34 as the only exception. Dickkopf‐1 was significantly associated with EMT‐related proteins (E‐cadherin, vimentin, Twist, and Slug), CSC‐related proteins (CD44 and nestin), VM‐related proteins (VE‐cadherin, MMP2 and MMP9) and β‐catenin nuclear expression (*P* < 0.05). No statistically significant relationship was found between EpCAM, CD34, and DKK1 (*P* > 0.05, Table 1, Fig. 2).

### Effects of DKK1‐transfection on EMT‐related proteins of NSCLC cells *in vitro*

The expression of DKK1 was detected among three lung cancer lines representing distinct histological entities, namely, H460 (large cell lung cancer), H1299 (lung adenocarcinoma) and A549 (lung adenocarcinoma). Based on Western blot results, H460 cells were selected as the lowest expression of DKK1, while A549 cells demonstrated the lowest highest levels (Fig. S1A). Following transfection, DKK1 expression was evaluated by western blotting to confirm the transfection efficiency (Fig. S1A). The effect of DKK1 on VM formation was also investigated.

**Figure 3:**
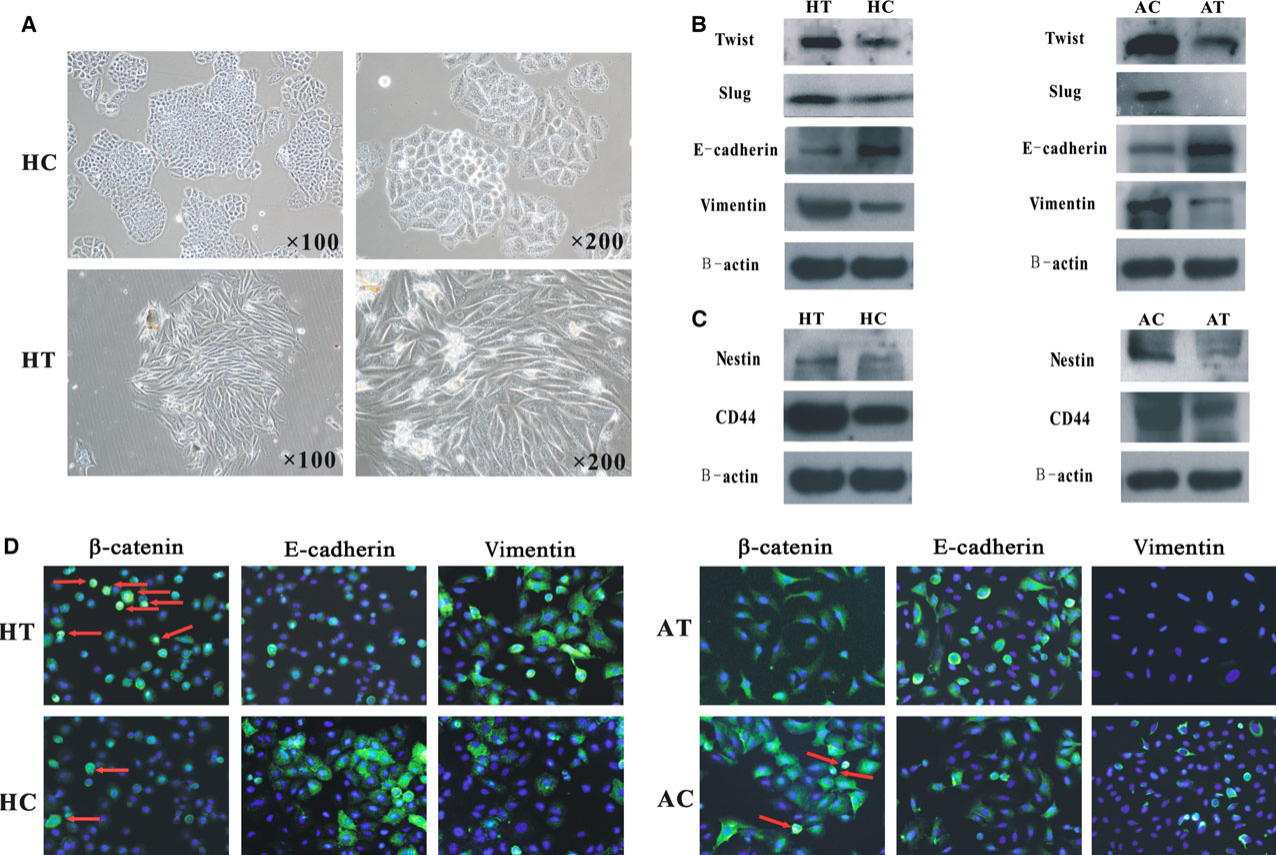
Differences between transfection group and the control (HT: H460transfaected with a pcDNA3.1‐DKK1 vector; HC: H460 cells transfected with an empty vector; AT: A549 cells silenced by a DKK1‐targeting siRNA; AC: A549 cells transfected with a non‐targeting siRNA). (**A**) Morphological appearances between HT cells and HC cells. HC cells were polygonal and cobble‐stone‐like, whereas HT cells were spindle shaped. (**B**) Changes in EMT‐related proteins following transfection. (**C**) Diversities of expression of CSC‐associated markers in transfected NSCLC cells. HT cells developed and AT cells lost more CSC features. (**D**) Immunofluorescence analysis showed changes in β‐catenin, E‐cadherin and vimentin expressions between transfection and control groups (×400). Both (**B**) and (**D**) showed that HT cells underwent EMT, whereas AT cells underwent mesenchymal–epithelial transition.

During stable transfection of the DKK1 plasmid into H460 cells, remarkable morphological changes were observed. H460 cells are typically polygonal and cobble‐stone‐like, which are features of epithelial phenotypes. Following transfection with DKK1, the cells gradually transformed into a spindle shape, which is a mesenchymal phenotype (Fig. 3A). Moreover, increased expressions of vimentin, Slug and Twist were detected, while expression of E‐cadherin, which is an epithelial marker, decreased (Fig. 3B and D).

By contrast, A549 cells exhibited more molecular characteristics of epithelial cells following transfection of DKK1‐shRNAinto these cells. E‐cadherin expression increased in the transfected cells, whereas protein levels of vimentin, Slug, and Twist were reduced (Fig. 3B and D).

### Effects of DKK1 overexpression/deletion on CSC‐related proteins of NSCLC cells *in vitro*

Based on our characterization of human primary tumours, the expression of CSC‐related proteins that were significantly associated with VM and DKK1 were examined. CD44 and nestin were both overexpressed in H460‐DKK1 cells and down‐regulated in A549‐siDKK1 cells (Fig. 3C). The expression of β‐catenin was also investigated in these transfected cells. We found that β‐catenin expression decreased in A549‐siDKK1 cells and increased in H460‐DKK1 cells (Fig. 5B and D). The immunofluorescent staining for β‐catenin confirmed the result of the western blot, and it also indicated that more β‐catenin located in the nuclei of H460‐DKK1 cells (Fig. 3D).

### Effects of DKK1 overexpression/silencing on migration and invasion capability of NSCLC cells *in vitro*

**Figure 4:**
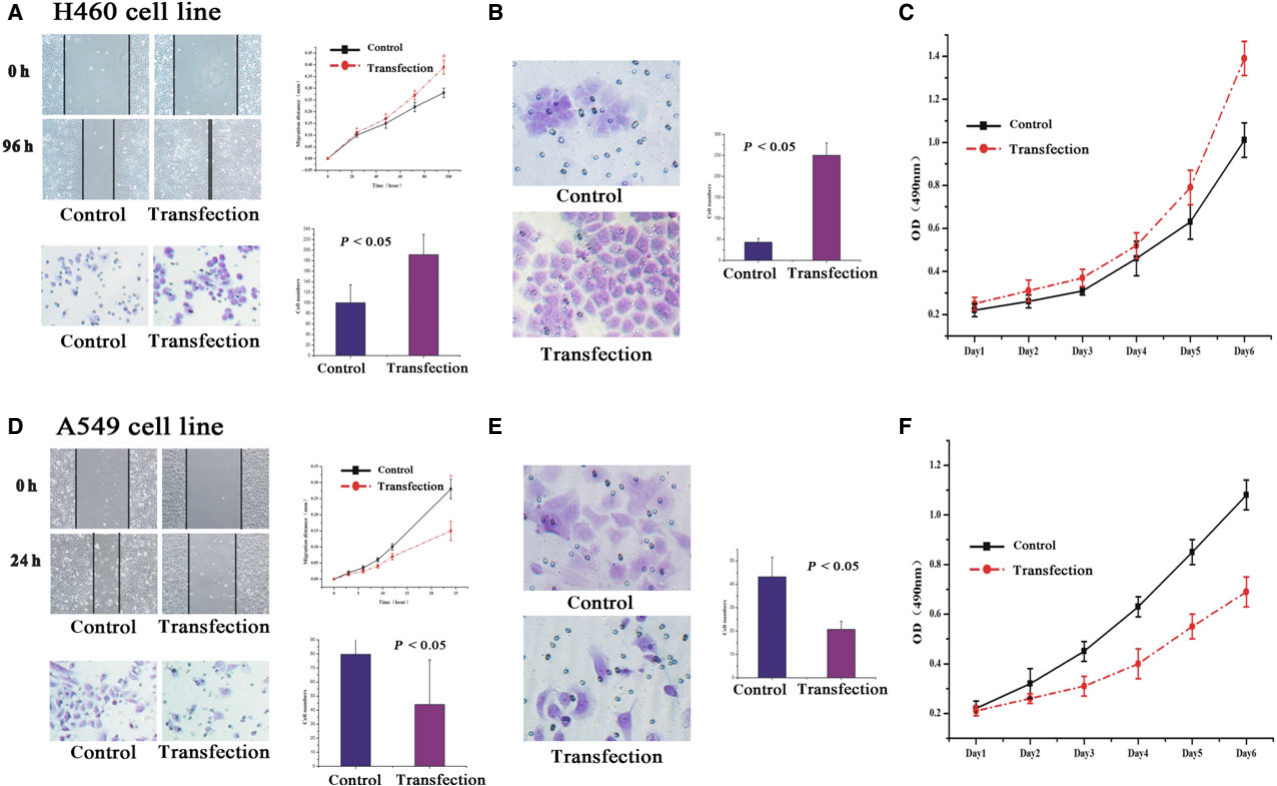
Changes in migration, invasion and proliferation abilities following DKK1 transfection into H460 cells and sh‐DKK1 in A549 cells. (**A**) Both wound‐healing and transwell assays without Matrigel demonstrated that transfected H460 cells migrate more readily than the control (*P* < 0.05). (**B**) Invasion number of H460‐DKK1 cells was higher than the control in Matrigel transwell assay (*P* < 0.05). (**C**) H460‐DKK1 cells proliferated quicker than the cells in the control group in the MTT assay (*P* < 0.05). Accordingly, transfected A549 cells showed less (**D**) migration, (**E**) invasion, and (**F**) proliferation abilities (*P* < 0.05) compared with the control.

To investigate the impact of DKK1 on migration, a wound‐healing assay and a transwell assay without Matrigel were both performed. In the wound‐healing assay, H460‐DKK1 cells migrated more rapidly than H460 cells, and the migration distances were 0.39 and 0.28 mm respectively (*P* < 0.05, Fig. 4A). Moreover, the cell numbers were higher for H460‐DKK1 cells that were passed, as compared to controls (191 *versus* 100, *P* < 0.05) (Fig. 4A). In the Matrigel transwell assay, the passed cells were also fixed, stained and counted. There was approximately a fivefold increase in cell invasion observed in the H460‐DKK1group compared with controls (*P* < 0.05, Fig. 4B). In keeping with these results, A549‐siDKK1 cells showed lower migration and invasion abilities than the control (*P* < 0.05, Fig. 4D and E).

### Abnormal expression of DKK1 regulated the proliferation ability of NSCLC cells *in vitro*

To evaluate the impact of DKK1 on proliferation, an MTT assay was performed. As expected, we observed increase in the growth rate of H460 cells overexpressing DKK1 (*P* < 0.05, Fig. 4A–C). Moreover, suppression of growth was also found as early as the fourth day in A549‐siDKK1 cells (*P* < 0.05, Fig. 4B and C).

### Effects of DKK1 overexpression/deletion on VM formation of NSCLC cells *in vitro*

**Figure 5:**
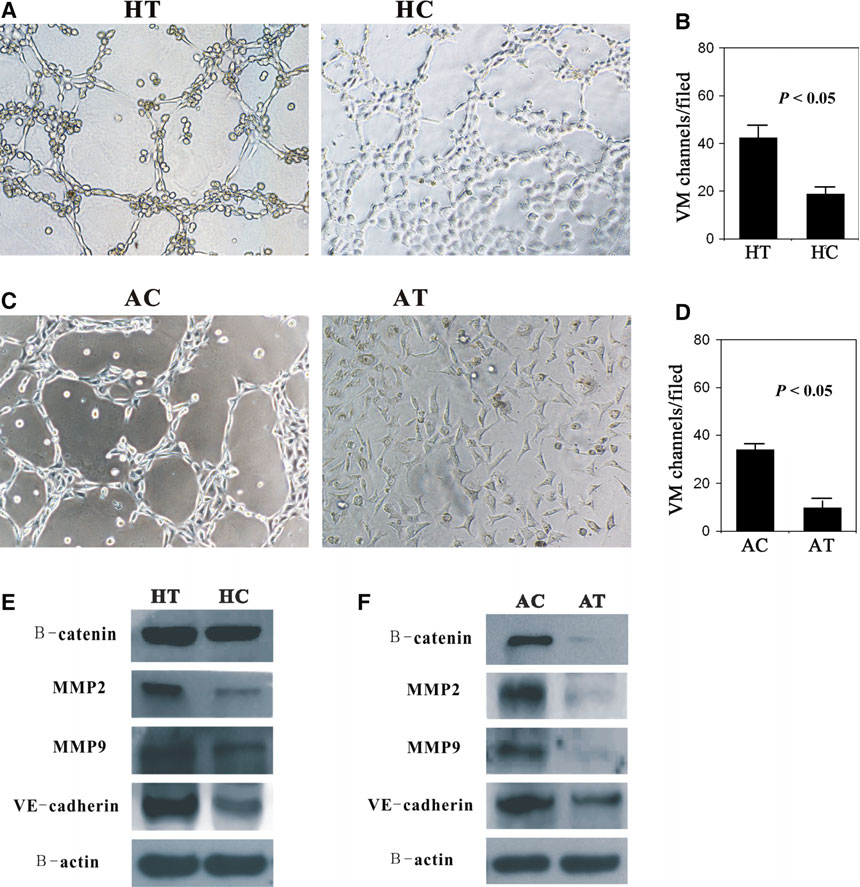
Varieties of VM formation between transfection and control groups (HT: H460transfaected with a pcDNA3.1‐DKK1 vector; HC: H460 cells transfected with an empty vector; AT: A549 cells silenced by a DKK1‐targeting siRNA; AC: A549 cells transfected with a non‐targeting siRNA). (**A** and **C**) Compared with the control, H460‐DKK1 cells formed more typical pipe‐like structures, and the inner walls of these tubes were smoother, whereas A549‐siDKK1 cells did not form these structures (×200). (**B** and **D**) Quantification of VM channels in each group. (**E** and **F**) VM‐related proteins (MMP2, MMP9 and VE‐cadherin) and β‐catenin were overexpressed in HT and obviously reduced in AT.

We evaluated the formation of VM in NSCLC cells using a 3D culture assay model. Compared with the control group, there were more pipe‐like structures that formed in the H460‐DKK1 group, and the inner walls of these pipes were smoother (Fig. 5A and B). Moreover, as shown in Figure 5C and D, A549 cells were unable to form channels following transfection with DKK1‐shRNA. By contrast, the control group had numerous pipeline structures.

Vasculogenic mimicry‐related proteins were also evaluated following DKK1 overexpression or suppression. In agreement with the results of the 3D culture, MMP2, MMP9 and VE‐cadherin expressions increased in the H460‐DKK1 group and decreased in the A549‐siDKK1 group compared with their corresponding controls (Fig. 5E and F).

### Effects of DKK1‐transfection on xenograft *in vivo*

**Figure 6:**
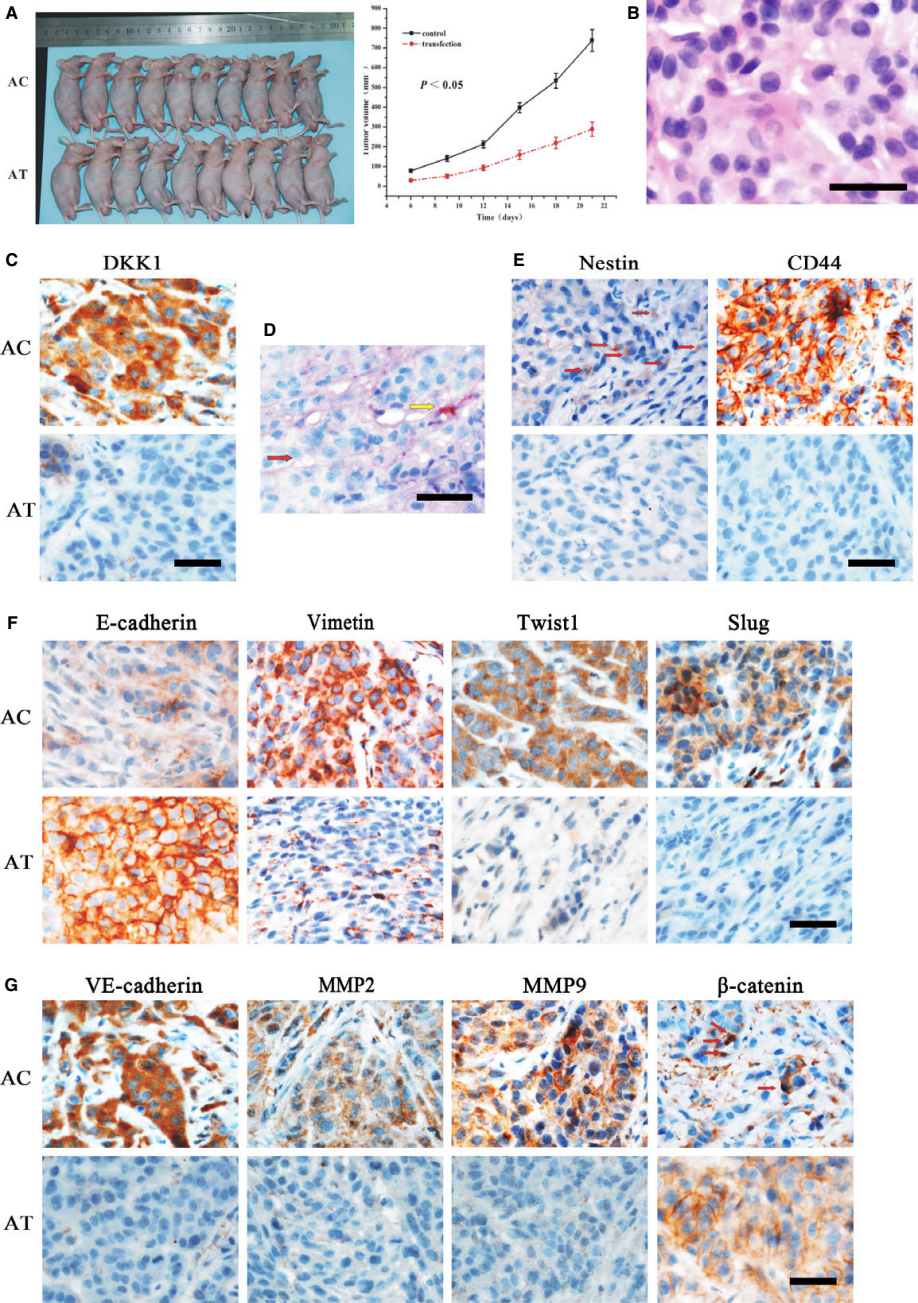
Effects of siDKK1‐transfection on xenograft (AC, A549 Group; AT, A549‐siDKK1 Group). (**A**) Xenografts in the AT group developed more slowly than those in the AC group (*P* < 0.05). (**B**) Hematoxylin and eosin staining of xenografts. (**C**) Xenografts in AT showed reduced DKK1‐expression than the control, which further confirmed the effect of transfection. (**D**) Results of endomucin/PAS double‐staining. The VM channel was positively expressed for PAS but negatively expressed for endomucin (red arrow). The endothelial channel was positively expressed for both endomucin and PAS (yellow arrow). (**E**) Nestin and CD44 were significantly down‐regulated in xenografts of AT, suggesting that AT cells lost CSC features. (**F**) Xenografts in AT showed up‐regulation of E‐cadherin and down‐regulation of vimentin, Slug, and Twist, indicating that AT cells experienced reverse EMT. (**G**) Xenografts in AT showed the attenuating abilities of VM formation by decreased expression of VE‐cadherin, MMP2 and MMP9. β‐catenin was also expressed in the membrane of AT cells, whereas a more positive expression was found in the nuclei of AC cells (red arrows), bars: 50 μm.

To further validate the effect of DKK1 on NSCLC cells, xenograft mouse models were performed. Following the subcutaneous transplantation of H460‐DKK1, A549‐siDKK1 and their corresponding control cells in nude mice, xenografts were established for all 40 mice. Compared to the corresponding control group, we found that transplanted tumour cells in H460‐DKK1 group grew more rapidly, and the tumours in A549‐siDKK1 group developed at a slower rate (*P* < 0.05, Fig. S2A and Fig. 6A). The tumour volume of the H460‐DKK1 group was larger than that in parental H460 cells (1736.09 and 841.60 mm^3^) respectively (*P* < 0.05, Fig. S2A). By contrast, the tumour size in the A549‐siDKK1 group (268.95 mm^3^) was smaller than that in the control (533.85 mm^3^) (*P* < 0.05, Fig. 6A).

Vasculogenic mimicry was also examined in seven of the mice in the H460‐transfection group, and was found to be higher than that in the control group (4/10, *P* < 0.05, Fig. S2G). Vasculogenic mimicry was found in two mice in the A549‐transfection group, and was lower than that in the A549‐control group (5/10, *P* < 0.05, Fig. 6G).

Moreover, compared to the corresponding control, E‐cadherin expression decreased in the H460‐DKK1 group and increased in the A549‐siDKK1 group (*P* < 0.05, Fig. 6, Figs S2–S4). The expression of other related proteins (vimentin, Twist, Slug, CD44, nestin, VE‐cadherin, MMP2, MMP9 and β‐catenin nuclear) increased in the H460‐DKK1 group and decreased in the A549‐siDKK1 group (*P* < 0.05, Fig. 6, Figs S2–S4).

## Discussion

Vasculogenic mimicry has been identified in several malignant tumours, including HCC, glioblastoma and breast cancer 17, 18, 19, 20. Based on these studies, VM results in more aggressive cancer and is associated with a poor prognosis. We found similar findings, in NSCLC specimens through the identification of VM which was associated with poor differentiation, metastasis, advanced stage, and a shorter survival period. With the unique structure of VM, tumour cells are exposed directly to the blood flow, which enable them to enter the microcirculation and metastasize to other organs with greater ease. In addition, VM most often occurs in large cell lung cancer, followed by lung adenocarcinoma and squamous cell carcinoma. Large cell lung cancer is the most pernicious subtype, which may have contributed to this result. We also evaluated the expression of DKK1 in human NSCLC tissues, and found that DKK1 was related with histological classification and differentiation similar to that in VM. Moreover, overexpression of DKK1 positively correlated with the existence of VM and high expression of some VM‐related proteins (MMP2, MMP9 and VE‐cadherin).

Several studies have shown that VM is usually regarded as an example of aggressive tumour cells with remarkable differentiation plasticity 20, 21. In this process, more aggressive tumour cells transdifferentiated, altered their cell markers, acquired more embryonic stem characteristics, and acted as endothelium cells to form the VM structure. Therefore, we can assume that the actions of EMT and CSC may be involved in VM formation.

Epithelial‐mesenchymal transition describes a series of events during which epithelial cells lose many of their epithelial characteristics and take on properties typical of mesenchymal cells, implying complex changes in cell architecture and behaviour 22, 23. Wnt signalling pathway is essential to the EMT induction. Cell division cycle 6 (Cdc6), a component of Wnt signalling pathway, has been shown to inhibit the translational activity of E‐cadherin 24. Overexpression of Cdc6 can suppress E‐cadherin and lead to EMT in a lung cancer cell line 24. Dickkopf‐1 is also a regulator of Wnt signalling pathway. Our data highlight a correlation between the existence of VM, overexpression of DKK1 and decreased E‐cadherin expression, and increased vimentin expression in NSCLC tissues. Moreover, overexpression of DKK1 in lung tumour cells decreased E‐cadherin expression and increased vimentin expression. β‐catenin, the central molecule of the Wnt pathway, and its nuclear expression, which has been shown to induce EMT and is used as a mesenchymal marker, was also positively related to VM and DKK1. Furthermore, VM and DKK1 demonstrated a correlation with Slug and Twist, which are two important positive regulators of EMT. Results of *in vitro* and *in vivo* experiments further indicate that DKK1 can sufficiently induce EMT and promote VM formation.

The CSC theory may explain the biological heterogeneity of solid tumours 25. Similar to normal adult stem cells, cancer stem cells possess high tumorigenic ability, as well as self‐renewal and pluripotency that could differentiate into different cell types 26. Our data suggest that CSC phenotype was associated with VM and concomitantly with DKK1 overexpression. Our *in vivo* studies also showed that DKK1‐overexpression in lung cancer cells exhibited CSC phenotype and were more aggressive. These xenograft tumours were more vascular and displayed more VM than cells from normal lung cancer cells. Cancer stem‐like cell may also directly contribute to tumour angiogenesis by converting into endothelial cell 18, 27.

Previous studies have reported that the cells engaged in VM may come from cancer cells endowed with transdifferentiation and stem‐cell plasticity, and may represent an incomplete differentiation of CSC towards endothelial lineage 18, 20, 28, 29. Smadja *et al*. also found that DKK1 enhanced angiogenic properties of endothelial colony‐forming cells *in vitro* and was required for endothelial colony‐forming cell and mesenchymal stem cell angiogenic phenotypes *in vivo* in breast cancer 30. Based on previous studies and our data, we postulate that overexpression of DKK1 can lead to transdifferentiation of lung cancer cells, resulting in the loss of their epithelial cell phenotypes and the occurrence of more mesenchymal cell phenotypes. Meanwhile, in the EMT process, these tumour cells also develop cancer stem cell features, which enhance their abilities of proliferation, invasion, migration and tumorigenesis. These cells can act as endothelium cells, which are known as mesenchymal cells, to form the VM channel. Thus, the tumour with VM shows more aggressive behaviour, can metastasize more easily, and leads to poorer prognosis. These CSCs are assumed to generate erythroid cells, as Zhang reported 31. Dickkopf‐1 seems to have opposing roles in different tissues and types of tumours. Similar to DKK1, the functions of the E2F transcription factors can vary significantly in malignancies of the digestive system 32. Previously, Qi *et al*. in our laboratory found that DKK1 inhibited EMT and VM in colon cancer. Considering the conflicting results in our laboratory, we speculate that the differences in results were because of the use of colon cancer cells in the previous study 8, whereas we used NSCLC 8. Ultimately, the role of DKK1 in cancer is not completely understood 33. Dickkopf‐1 can bind to the Wnt co‐receptor LRP5/6 and transmembrane proteins Kremen1/2 to form a tripolymer that block the binding of Wnt, LRP5/6 and Fz receptor to cause the degradation of β‐catenin 34. Hence, DKK1is able to inhibit the β‐catenin‐mediated transcription of the EMT‐related molecule including snail, slug and twist1 in breast cancer, melanoma and colon cancer 35, 36, 37. Interestingly, DKK1 is also a downstream target of Wnt signalling 33, and thus there is a negative feedback loop that activation of the canonical Wnt signalling (but not non‐canonical Wnt signalling) which causes up‐regulation of DKK1 expression 10. The DKK1 promoter can be transactivated by the β‐catenin/ T‐cell factor (TCF) complex 38. Dickkopf‐1 is overexpressed in human hepatoblastomas, multiple myeloma, Wilms' tumours and oesophageal carcinomas, which display unregulated activation of Wnt signalling 11, 33.

In conclusion, this study describes the previously unrecognized role of DKK1 and authenticates the hypothesis that DKK1 promotes VM formation by inducing EMT‐related proteins and by developing CSC characteristics in NSCLC. Our findings may benefit future studies on the mechanism of NSCLC progression and provide new hope for NSCLC diagnosis and therapeutic strategies.

## Funding

This work was supported by grants from the Key project of the National Natural Science Foundation of China (No. 81230050), the National Natural Science Foundation of China (No. 81172046, 81572872) and the Key project of the Tianjin Natural Science Foundation (No. 12JCZDJC23600).

## Conflict of interest

There is no any conflict of interests.

## Author contribution

Danfang Zhang and Baocun Sun were responsible for the conception, design and final approval of the manuscript to be published. Lingli Yao was involved in the design, animal experiment, and drafting of the manuscript. Xiulan Zhao was responsible for conception, IHC staining, PAS/CD31 double staining and taking photographs. Yanrong Liu and Xueming Zhao collected the data of patients with lung cancer. Qiang Gu and Na Che made the sections and tissue blocks for the mice model. Yanhui Zhang and Yanjun Zheng were participated in the animal treatment and data collection during the animal experiment. Fang Liu performed western blot and participated in the animal experiment. Yong Wang and Jie Meng were responsible for cell culture. All authors read and approved the final manuscript.

## Supporting information


**Figure S1** Detection of DKK1 expression by western blot. (**A**) Three NSCLC cell lines, A549, H460 and H1299, are used to detect the expression of DKK1 in this study. Western blot shows that A549 cells expressed highest level of DKK1, and the level of DKK1 expression in H460 is lowest in three cell lines. (**B** and **C**) H460 cells are selected for overexpression of DKK1, and A549 cells are selected for down‐regulation of DKK1. After transfection, DKK1 expression is detected by western blot to confirm the transfection efficiency. HT: H460 transfaected with a pcDNA3.1‐DKK1 vector; HC: H460 cells transfected with an empty vector; AT: A549 cells silenced by a DKK1‐targeting siRNA; AC: A549 cells transfected with a non‐targeting siRNA.

**Figure S2** Effects of DKK1‐transfection on xenograft (HT: H460‐DKK1 group; HC: H460 control group). (**A**) Xenografts showed higher rate of tumour growth in the HT group compared with the HC group (*P* < 0.05). (**B** and **D**) Hematoxylin and eosin staining and endomucin/PAS double‐staining. Red arrow showed that the VM channel and yellow arrow showed an endothelial vessel, which was further demonstrated by endomucin/PAS double‐staining in (**D**). (**C**) Xenografts in HT showed increased DKK1‐expression than the control, which also confirmed the effect of transfection. (**E**) Expressions of nestin and CD44 were significantly augmented in xenografts of HT, and HT cells acquired CSC features. (**F**) Xenografts in HT showed EMT by the down‐regulation of E‐cadherin and up‐regulation of vimentin, Slug and Twist. (**G**) VE‐cadherin, MMP2 and MMP9 were increasingly expressed in transplanted tumours of HT, which indicated the fortified abilities of VM formation. β‐catenin nuclear expression also increased in HT tumours, bars: 50 μm.

**Figure S3** Quantifications of the expression of CSC‐related and VM‐related proteins in the A549 Control Group (AC) and the A549‐siDKK1 Group (AT). (**A**) Quantifications of the expression of DKK1, Nestin and CD44. (**B**) Quantifications of the expression of E‐cadherin, vimentin, Twist and Slug. (**C**) Quantifications of the expression of VE‐cadherin, MMP2, MMP9 and β‐catenin‐nu. Error bar: standard deviation (S.D.).

**Figure S4** Quantifications of the expression of CSC‐related and VM‐related proteins in the H460‐DKK1 group (HT) and H460 control group (HC). (**A**) Quantifications of the expression of DKK1, Nestin and CD44. (**B**) Quantifications of the expression of E‐cadherin, vimentin, Twist and Slug. (**C**) Quantifications of the expression of VE‐cadherin, MMP2, MMP9 and β‐catenin‐nu. Error bar: standard deviation (S.D.).

**Table S1** Correlation among VM, DKK1 and clinicopathological features of NSCLC.

**Table S2** Information of primary antibodies used in this study.
