# Supplementary figures and images for: Dickkopf‐1‐promoted vasculogenic mimicry in non‐small cell lung cancer is associated with EMT and development of a cancer stem‐like cell phenotype
Source: J Cell Mol Med. 2016 May 31;20(9):1673–85. doi: 10.1111/jcmm.12862 (PMC4988283; doi:10.1111/jcmm.12862)

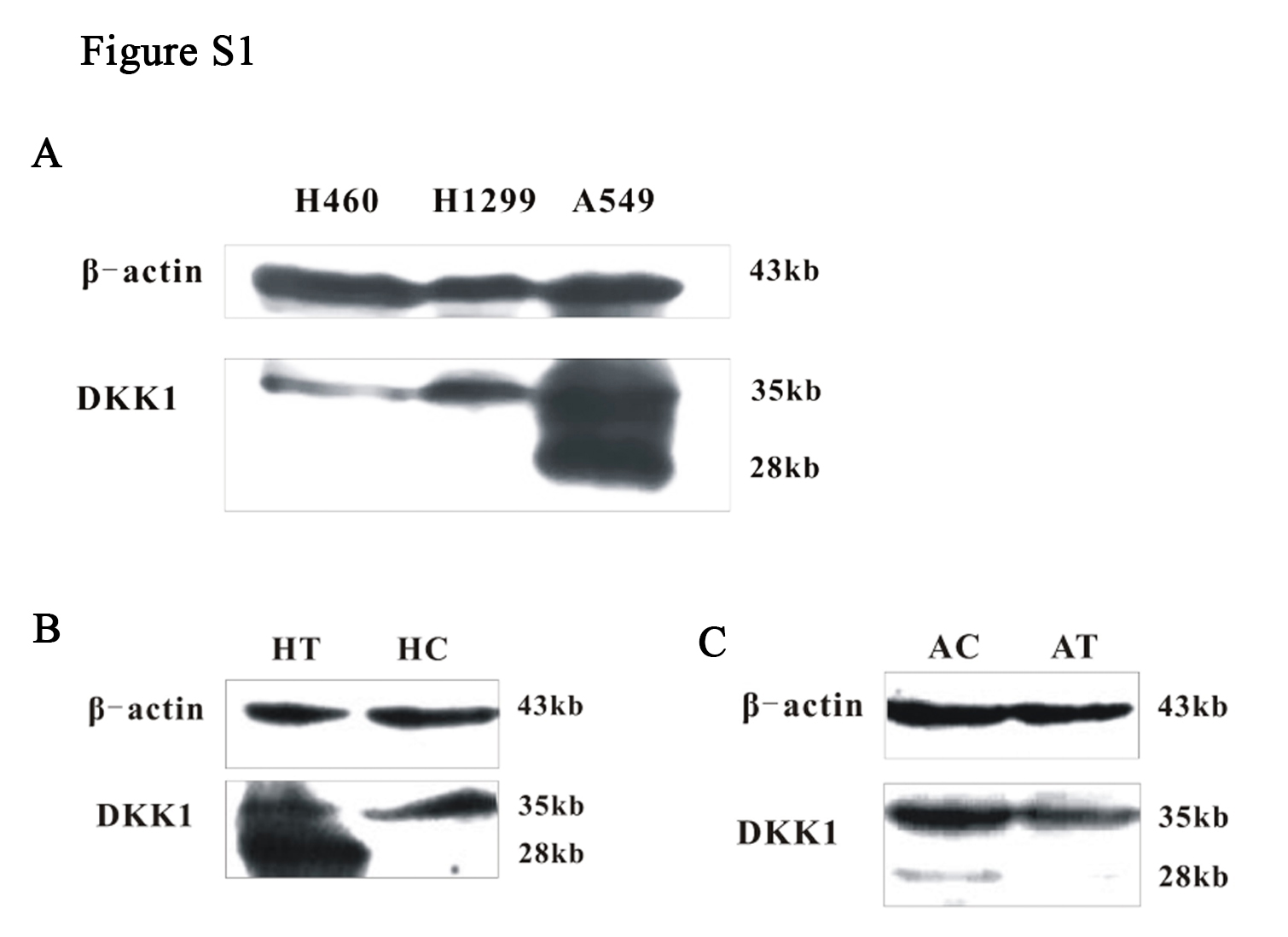

Supplement: Supplementary file 1 — Figure S1 Detection of DKK1 expression by western blot. (A) Three NSCLC cell lines, A549, H460 and H1299, are used to detect the expression of DKK1 in this study. Western blot shows that A549 cells expressed highest level of DKK1, and the level of DKK1 expression in H460 is lowest in three cell lines. (B and C) H460 cells are selected for overexpression of DKK1, and A549 cells are selected for down‐regulation of DKK1. After transfection, DKK1 expression is detected by western blot to confirm the transfection efficiency. HT: H460 transfaected with a pcDNA3.1‐DKK1 vector; HC: H460 cells transfected with an empty vector; AT: A549 cells silenced by a DKK1‐targeting siRNA; AC: A549 cells transfected with a non‐targeting siRNA. [file JCMM-20-1673-s001.jpg]

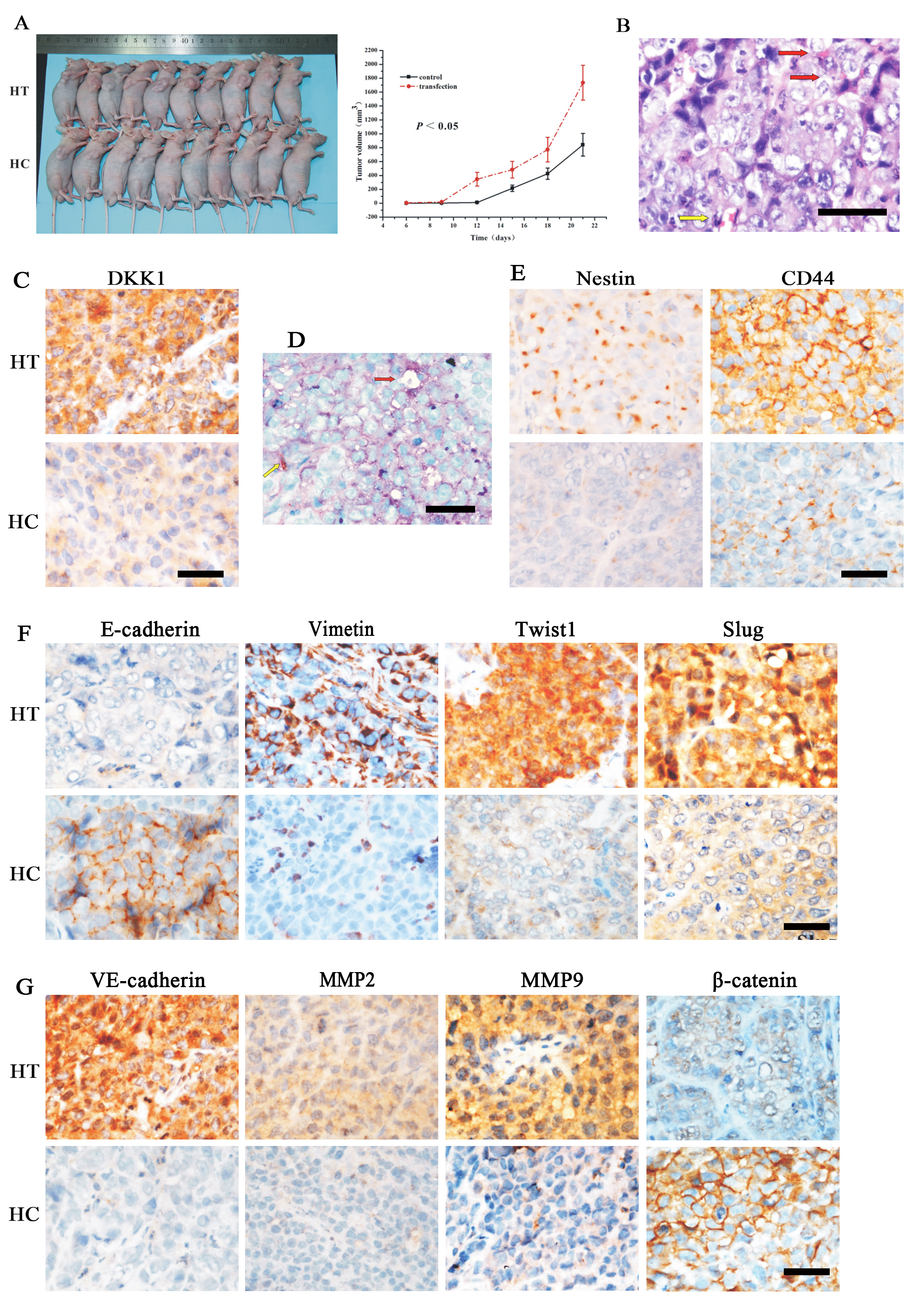

Supplement: Supplementary file 2 — Figure S2 Effects of DKK1‐transfection on xenograft (HT: H460‐DKK1 group; HC: H460 control group). (A) Xenografts showed higher rate of tumour growth in the HT group compared with the HC group (P < 0.05). (B and D) Hematoxylin and eosin staining and endomucin/PAS double‐staining. Red arrow showed that the VM channel and yellow arrow showed an endothelial vessel, which was further demonstrated by endomucin/PAS double‐staining in (D). (C) Xenografts in HT showed increased DKK1‐expression than the control, which also confirmed the effect of transfection. (E) Expressions of nestin and CD44 were significantly augmented in xenografts of HT, and HT cells acquired CSC features. (F) Xenografts in HT showed EMT by the down‐regulation of E‐cadherin and up‐regulation of vimentin, Slug and Twist. (G) VE‐cadherin, MMP2 and MMP9 were increasingly expressed in transplanted tumours of HT, which indicated the fortified abilities of VM formation. β‐catenin nuclear expression also increased in HT tumours, bars: 50 μm. [file JCMM-20-1673-s002.jpg]

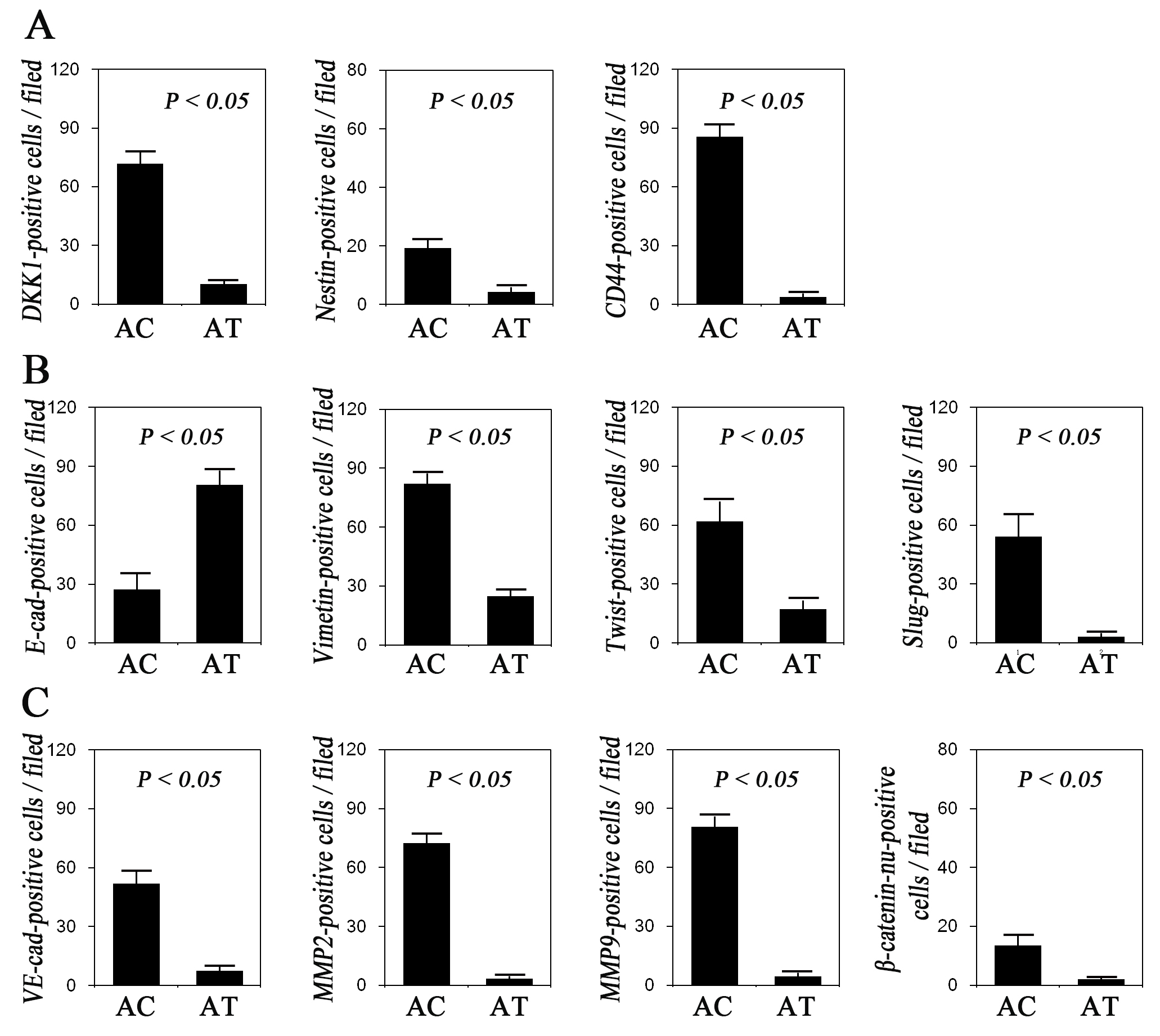

Supplement: Supplementary file 3 — Figure S3 Quantifications of the expression of CSC‐related and VM‐related proteins in the A549 Control Group (AC) and the A549‐siDKK1 Group (AT). (A) Quantifications of the expression of DKK1, Nestin and CD44. (B) Quantifications of the expression of E‐cadherin, vimentin, Twist and Slug. (C) Quantifications of the expression of VE‐cadherin, MMP2, MMP9 and β‐catenin‐nu. Error bar: standard deviation (S.D.). [file JCMM-20-1673-s003.jpg]

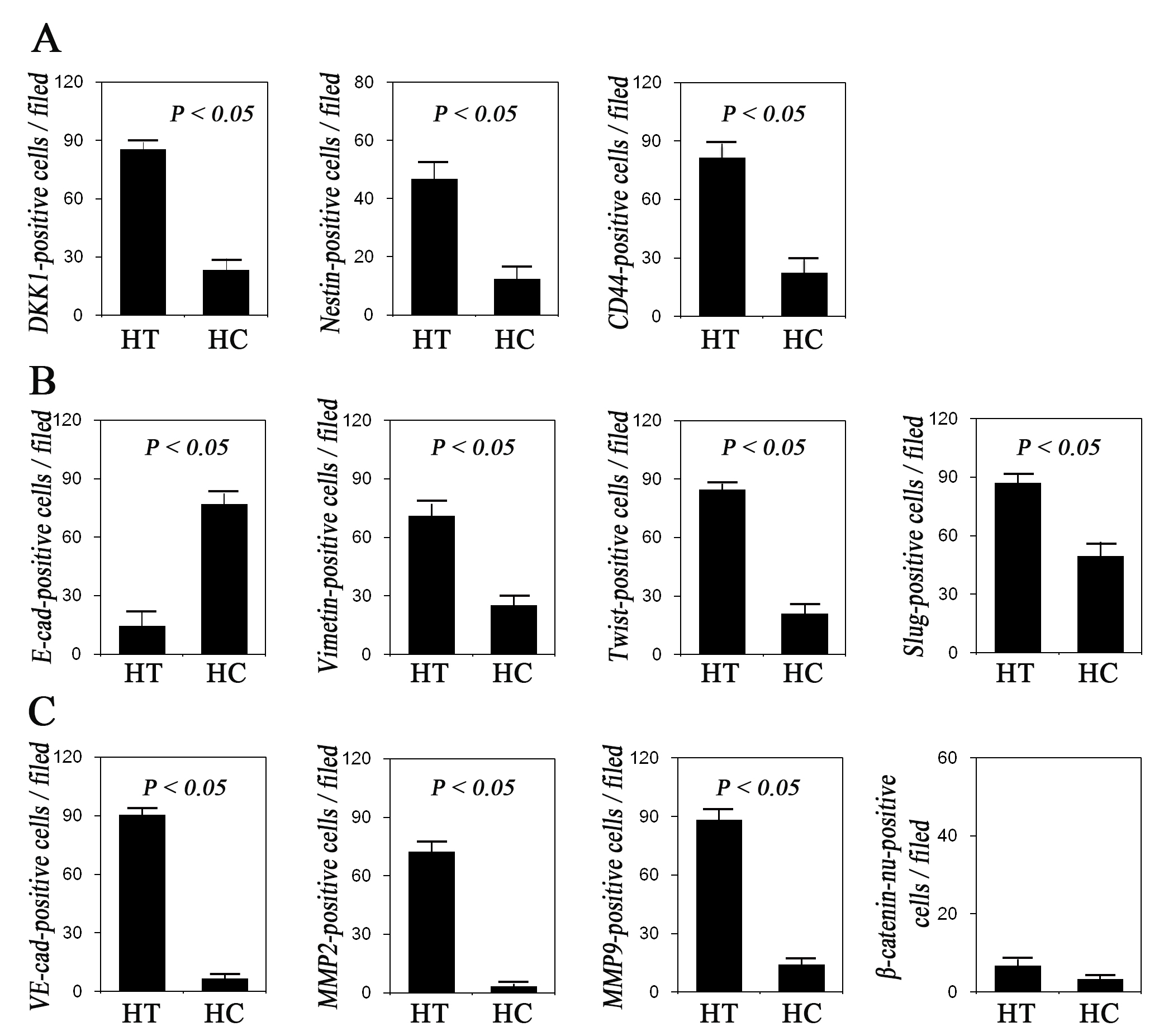

Supplement: Supplementary file 4 — Figure S4 Quantifications of the expression of CSC‐related and VM‐related proteins in the H460‐DKK1 group (HT) and H460 control group (HC). (A) Quantifications of the expression of DKK1, Nestin and CD44. (B) Quantifications of the expression of E‐cadherin, vimentin, Twist and Slug. (C) Quantifications of the expression of VE‐cadherin, MMP2, MMP9 and β‐catenin‐nu. Error bar: standard deviation (S.D.). [file JCMM-20-1673-s004.jpg]
