# Supplementary material for: Dickkopf‐1‐promoted vasculogenic mimicry in non‐small cell lung cancer is associated with EMT and development of a cancer stem‐like cell phenotype
Source: J Cell Mol Med. 2016 May 31;20(9):1673–85. doi: 10.1111/jcmm.12862 (PMC4988283; doi:10.1111/jcmm.12862)
Supplement: Supplementary file 5 — Table S1 Correlation among VM, DKK1 and clinicopathological features of NSCLC. [file JCMM-20-1673-s005.doc]

Table S1. Correlation between VM, DKK1, and clinicopathological features of NSCLC

| Variant | | N | VM | | *P*-value | DKK1 | | *P*-value |
| --- | --- | --- | --- | --- | --- | --- | --- | --- |
|  |  |  | Negative | Positive |  | Negative | Positive |  |
| Gender | |  |  |  |  |  |  |  |
|  | Male | 145 | 122 | 23 | 0.184 | 46 | 99 | 0.147 |
|  | Female | 60 | 55 | 5 |  | 26 | 34 |  |
| Age (yr) | |  |  |  |  |  |  |  |
|  | ＜60 | 101 | 86 | 15 | 0.687 | 34 | 67 | 0.770 |
|  | ≥60 | 104 | 91 | 13 |  | 38 | 66 |  |
| Size (cm) | |  |  |  |  |  |  |  |
|  | ＜5 | 107 | 92 | 15 | 1.000 | 38 | 69 | 1.000 |
|  | ≥5 | 98 | 85 | 13 |  | 44 | 64 |  |
| Location | |  |  |  |  |  |  |  |
|  | Center | 106 | 91 | 15 | 0.842 | 36 | 70 | 0.770 |
|  | Peripheral | 99 | 86 | 13 |  | 36 | 63 |  |
| Histological classification | | |  |  |  |  |  |  |
|  | SCC | 79 | 75 | 4 | <0.001 | 32 | 47 | 0.028 |
|  | AC | 75 | 68 | 7 |  | 30 | 45 |  |
|  | LCC | 51 | 34 | 17 |  | 10 | 41 |  |
| Differentiation | |  |  |  |  |  |  |  |
|  | Well | 35 | 35 | 0 | <0.001 | 17 | 18 | 0.008 |
|  | Moderate | 87 | 81 | 6 |  | 36 | 51 |  |
|  | Poor | 83 | 61 | 22 |  | 19 | 64 |  |
| Pleura invasion | |  |  |  |  |  |  |  |
|  | No | 113 | 96 | 17 | 0.547 | 42 | 71 | 0.557 |
|  | Yes | 92 | 81 | 11 |  | 30 | 62 |  |
| Lymph node metastasis | | |  |  |  |  |  |  |
|  | No | 117 | 104 | 13 | 0.227 | 44 | 73 | 0.460 |
|  | Yes | 88 | 73 | 15 |  | 28 | 60 |  |
| T stage |  |  |  |  |  |  |  |  |
|  | T1+T2 | 149 | 134 | 15 | 0.021 | 57 | 92 | 0.142 |
|  | T3+T4 | 56 | 43 | 13 |  | 15 | 41 |  |
| Clinical stage | |  |  |  |  |  |  |  |
|  | Ⅰ+Ⅱ | 158 | 144 | 14 | 0.001 | 58 | 100 | 0.487 |
|  | Ⅲ+Ⅳ | 47 | 33 | 14 |  | 14 | 33 |  |
| Distant metastasis | |  |  |  |  |  |  |  |
|  | No | 147 | 136 | 11 | <0.001 | 54 | 93 | 0.517 |
|  | Yes | 58 | 41 | 17 |  | 18 | 40 |  |
| Therapy before surgery | | |  |  |  |  |  |  |
|  | No | 186 | 160 | 26 | 1.000 | 65 | 121 | 1.000 |
|  | Yes | 19 | 17 | 2 |  | 7 | 12 |  |
| Therapy after surgery | | |  |  |  |  |  |  |
|  | No | 98 | 83 | 15 | 0.547 | 28 | 70 | 0.079 |
|  | Yes | 107 | 94 | 13 |  | 44 | 63 |  |
