# Supplementary material for: Dickkopf‐1‐promoted vasculogenic mimicry in non‐small cell lung cancer is associated with EMT and development of a cancer stem‐like cell phenotype
Source: J Cell Mol Med. 2016 May 31;20(9):1673–85. doi: 10.1111/jcmm.12862 (PMC4988283; doi:10.1111/jcmm.12862)
Supplement: Supplementary file 6 — Table S2 Information of primary antibodies used in this study. [file JCMM-20-1673-s006.doc]

Supplementary table 2. Information of primary antibodies used in this study

| Antibody | Source | NO. | Company | Dilution | Applications |
| --- | --- | --- | --- | --- | --- |
| DKK1 | Rabbit | SC-25516 | Santa cruz | 1:100 | IHC, WB |
| MMP2 | Rabbit | 10373-2-Ap | Luosai | 1:100 | IHC, WB |
| MMP9 | Goat | SC-6840 | Santa cruz | 1:200 | IHC, WB |
| VE-cadherin | Rat | 33168 | Abcam | 1:200 | IHC, WB |
| Twist 1 | Rabbit | sc-15393 | Santa cruz | 1:200 | IHC, WB |
| Slug | Rabbit | sc-166902x | Santa cruz | 1:50 | IHC, WB |
| E-cadherin | Rabbit | SC-7870 | Santa cruz | 1:400 | IHC, WB, IF |
| Vimetin | Rabbit | EPR3776 | Epitomics | 1:400 | IHC, WB, IF |
| β-catenin | Mouse | CAT-5H10 | Zymed | 1:100 | IHC, WB, IF |
| Nestin | Mouse | 10C2 | Zymed | 1:50 | IHC, WB |
| CD44 | Mouse | SC-53298 | Santa cruz | 1:200 | IHC, WB |
| EpCAM | Mouse | MOC31 | Zymed | Ready to use | IHC |
| CD31 | Mouse | 1A10 | Zymed | 1:60 | IHC |
| CD34 | Mouse | QBEnd/10 | Zymed | 1:200 | IHC |
| Endomucin | Rat | 11-5851-80 | eBioscience | 1:400 | IHC |
| β-actin | Rabbit | sc-130657 | Santa cruz | 1:1000 | WB |

IHC: immunohistochemistery; IF: immunofluorescence histochemical staining; WB: western blott
